# Brazilian Food Truck Consumers’ Profile, Choices, Preferences, and Food Safety Importance Perception

**DOI:** 10.3390/nu11051175

**Published:** 2019-05-25

**Authors:** Lígia Isoni Auad, Verônica Cortez Ginani, Eliana dos Santos Leandro, Elke Stedefeldt, Aline Costa Santos Nunes, Eduardo Yoshio Nakano, Renata Puppin Zandonadi

**Affiliations:** 1 Department of Nutrition, Faculty of Health Sciences, University of Brasilia (UnB), Campus Darcy Ribeiro, Asa Norte, Brasilia DF 70910-900, Brazil; vcginani@gmail.com (V.C.G.); elisanleandro@yahoo.com.br (E.d.S.L.); renatapz@yahoo.com.br (R.P.Z.); 2 GeQual—Study Group of Food Quality, Centro de Desenvolvimento do Ensino Superior em Saúde, Universidade Federal de São Paulo, São Paulo SP 04021-001, Brazil; elkesnutri@gmail.com; 3 Department of Pharmacy, Faculty of Health Sciences, University of Brasília (UnB), Campus Darcy Ribeiro, Asa Norte, Brasilia DF 70910-900, Brazil; alinecostasn@gmail.com; 4 Department of Statistics, Institute of Exact Sciences, University of Brasilia (UnB), Campus Darcy Ribeiro, Asa Norte, Brasilia DF 70910-900, Brazil; eynakano@gmail.com

**Keywords:** food truck, consumers, choices, preferences, food safety importance perception, Brazil

## Abstract

This study aimed to investigate food truck consumers’ profile, choices, preferences, and food safety importance perception. We conducted structured interviews with a convenient sample of 133 food truck consumers in the Federal District, Brazil. Most of the participating consumers were married (52%) and female (56%), who had completed at least tertiary school (81%). The interviews revealed that most food truck consumers eat from food trucks once or twice a week (96%), usually near home (74%), and have an average per capita expenditure of approximately US $5 to US $9.99 (70%). Hamburgers and sandwiches are the most popular food among consumers (72%). Consumers indicated that taste (30%) was the most important reason to choose a food truck and that poor vehicle hygiene (30%) was the main point assigned for not opting for a food truck. Food hygiene and vendors’ personal hygiene were considered important by consumers when eating from food trucks (78% and 80%, respectively). Considering all food truck consumers interviewed and the questions about food safety importance perception, the minimum score was 1 and the maximum was 2.9, with a mean score of 1.68 (SD = 0.46), indicating a high level of perceived importance. The instrument of food safety importance perception presented a Cronbach’s alpha coefficient of 0.73, indicating good internal consistency. No significant differences were observed in the food safety importance perception scores in gender (0.192), marital status (0.418), level of education (0.652) or food safety training (0.166). However, significant differences were found in the food safety importance perception scores for age (0.026) and the presence of children (0.001). The findings of this study indicate that there remains the need for consumers to comprehend their role in the food supply chain. Food safety and food handling practices are of public concern, and strategies are required to prevent foodborne diseases. Future public health interventions aiming to increase consumer knowledge and awareness of food safety should be emphasized.

## 1. Introduction

Over the past decades, the feeding profile of the population in both developed and developing countries has been changing due to the process of urbanization and globalization [1]. The lack of time for food preparation and consumption, along with the increasing demand for food diversification, availability, and accessibility, have led to an increase in the popularity of eating out of home [2]. The most recent data from the Pesquisa de Orçamentos Familiares (POF—Household Budget Survey) carried out by Brazilian Institute of Geography and Statistics (IBGE) shows that food purchase spent with food outside of the home in that country rose from 24.1% in 2002–2003 to 31.1% 2008–2009, representing a 30% growth in six years [3].

In parallel with the increase of food consumption outside home, the emerging industry of food trucks (FTs) is facing a rapid expansion and now represents one of the best performing segments in foodservice. In 2015, the U.S. FT industry was valued at US $856.7 million, with a forecast for 2020 of US $996.2 million [4]. According to statistics collected by Serviço Brasileiro de Apoio às Micro e Pequenas Empresas (SEBRAE—Brazilian Micro and Small Business Support Service), the annual revenue of FTs in Brazil in 2014 was 140 billion reais (about 35 billion USD). This industry is expected to continue thriving not only due to their economic and logistic advantages in comparison to brick and mortar restaurants—including low start-up costs, fewer permits and operational expenses, and lower maintenance levels—but also due to location variety, easier consumer outreach, and versatile food choices, which range from traditional/authentic cuisine to modern and sophisticated gourmet options, at affordable prices [5].

FTs are itinerant commercial kitchens, which are similar to brick and mortar restaurants—regarding the special attention needed on time and temperature control during the food process chain—and the street food (SF) vending sector at the same time—when considering their selling points and exposure to the environmental conditions [6]. In addition to offering fast meals in places of easy access and with fast services, FTs must ensure access to safe food considering the hygienic–sanitary perspective. However, the safety of the FT sector is of major concern, since these vehicles, due to their itinerant nature, generally escape effective food safety regulation and inspection [7]. A study performed in Brazil by Auad et al. [8] revealed that FTs had a high level of inadequacy regarding their hygienic–sanitary practices and conditions and a high rate of contaminated food samples, which raise the risk of foodborne diseases (FBDs) outbreaks.

FBDs remain a public health challenge despite the constant global effort from industries and governments to ensure the hygienic–sanitary quality of food production. According to the World Health Organization (WHO) ‘Estimates of the Global Burden of Foodborne Diseases’ [9], approximately 600 million cases of illness and 420,000 deaths in 2010 were caused by 31 foodborne hazards, including bacteria, viruses, parasites, toxins, and chemicals. In Brazil, the National Notifiable Diseases Information System (SINAN—Sistema de Informação de Agravos de Notificação) notified of a total of 6632 FBD outbreaks from 2007 to 2016 among 469,482 people who were exposed to the hazards during the same period [10].

Consumers have a right to expect that the foods they purchase and consume will be safe and of high quality from the sensory, nutritional, and microbiological points of view [11]. The establishment of effective strategies to prevent the contamination and the evaluation of the food production are fundamental to control the production process and provide safe food [12]. Food safety is a shared responsibility among entities, which means that along with farmers, manufacturers, and food handlers, consumers are responsible for ensuring that food is safe and suitable for consumption [13] and play an important role in preventing FBDs. While the food industry is responsible for implementing food safety standards and governments bear an obligation to monitor and enforce these standards, consumers must understand how to protect themselves against FBDs both in choice and handling of food. Consumers must also recognize and act on unsafe hygienic–sanitary practices and conditions of food establishments. 

Consumers’ consumption decisions involve balancing perceived benefits and risks [14]. Perceived benefit is defined as a consumer’s belief about the extent to which he/she will become better off from the purchase and positively affects his/her intention to purchase. On the other hand, perceived risk is defined as the potential uncertain negative outcomes from the purchase. This theoretical framework assumes that consumers make decisions to maximize the net valence resulting from the positives and negatives attributes of the decision [15]. According to the proposed theory, individuals tend to avoid a risky choice in the presence of gains and accept a risky choice in the presence of losses [16]. With respect to eating food outside home, consumers’ choices are usually guided by the perceived benefits—food sensory characteristics (such as taste), hedonic value and convenience—rather than the perceived risks—food safety issues [17]. In addition, consumers’ risk perception is influenced by the phenomenon of optimistic bias, or ‘unrealistic optimism’, supporting the belief that FBDs will not happen to them [18]. Therefore, low levels of perceived risk and/or high levels of perceived benefits from FT consumers may negatively influence their behavioral intention and attitude toward food purchase decisions due to underestimation of the risks associated with FT consumption and neglect of precautionary measures related to FBDs. Given the increasing presence of FTs in urban regions and the optimistic bias phenomenon related to food safety, this study aimed to investigate FT consumers’ profile, choices, preferences, and food safety importance perception.

## 2. Materials and Methods

This is a cross-sectional, quantitative, and exploratory study carried out in the Federal District of Brazil. FT consumers were conveniently sampled. We used a structured multiple-response questionnaire comprising 29 items (Supplementary file), which contained questions about consumers’ socioeconomic conditions (gender, age, marital status, level of education, monthly income), their preferences and perceptions when eating from FTs, including time and place of consumption, the reasons why they chose their food options, the frequency, cost, and company (with whom) when eating from FTs, and the reasons to choose or not to choose a FT. 

To evaluate the food safety importance perception of consumers, we elaborated 10 questions based on Brazilian national legislation for Good Practices [19], the General Principles of Food Hygiene of the Codex Alimentarius [20] and the Five Keys to Safer Food Manual [21]. Consumers were asked to evaluate 10 food safety importance perception questions, regarding their importance, using a five-point scale, as follows: (1) “Extremely important”; (2) “Very important”; (3) “Indifferent”; (4) “Slightly important”; and (5) “Not at all important”. The answers to the importance perception were classified as high perceived importance (1–2), average perceived importance (>2–<4) and low perceived importance (≥4–5) for each question. The overall importance perception score was defined based on the mean value of all 10 questions.

During this study, FTs were usually parked in specific locations during the night in clusters of between 6 to 10 vehicles, offering different menu options. One-hundred and thirty-three (*n* = 133) consumers agreed to participate in the interview. All interviews were conducted in Brazilian Portuguese, at the dining area of FTs during the consumption moment, and took 15–20 min to complete.

Descriptive statistics are presented as means and standard deviations for the quantitative variables and as frequencies and percentages for the categorical variables. All hypothesis tests were bicaudal with a 5% significance value. The statistical analysis considered the score of the 10-item food safety importance perception instrument. The response of each item of the instrument follows a scale of 1 to 5, and the overall importance perception score was defined as the mean value of these responses. Thus, the score of this questionnaire can vary between 1 and 5—the higher its value, the lower the perception of food safety. 

The internal consistency of the instrument was verified by the Cronbach’s alpha coefficient. All statistical analyses were performed using the IBM SPSS version 22.0 software (SPSS Inc., Chicago, IL, USA). Comparison of the scores according to age group, gender, marital status, presence of children, level of education, and food safety training was performed as follows. A possible difference between the scores of the instrument among the variables considered was performed using *t*-tests when the variable presented only two categories and by means of ANOVA followed by Tukey’s post-hoc when the variable had three or more categories. Normality of the instrument scores was verified by the Kolmogorov–Smirnov Test.

The ethical and methodological aspects of this study had the approval by the Ethics Commission of the University of Brasília (UnB), registered under number CEP no. 2.178.214. We obtained written informed consent of all participants and assured their anonymity and confidentiality throughout the study.

## 3. Results

Table 1 summarizes the demographic characteristics of FT consumers, the sample distribution according to the sociodemographic characteristics considered in the study, and the association of those variables with the food safety importance perception scores of the consumers.

The Cronbach’s alpha coefficient was used for the determination of internal consistency. Cronbach’s alpha values range from 0 to 1, with values near to 0 meaning no reliability and values near to 1 indicating perfect reliability. Cronbach’s alpha coefficient above 0.7 is considered satisfactory [22]. The food safety importance perception instrument presented a Cronbach’s alpha coefficient of 0.73, indicating good internal consistency.

Table 2 shows the results of the survey concerning consumers’ choices and preferences towards FTs.

Table 3 displays the results concerning the food safety importance perception of consumers. This table provides more insight into for which food safety aspects, the importance perception of FT consumers is lower or higher.

## 4. Discussion

The sociodemographic profile in our study showed that most of the consumers were young females (average age 35.6 ± 10.84 years, ranging from 17 to 69 years), with high educational level and no food safety training. These findings concur with those of the earlier study of Samapundo et al. [23], which showed that most SF consumers from Vietnam were females, with an average age of 29.7 ± 10.9 years, who had completed the tertiary school and had not received any food safety training. The profile of FT consumers revealed in our study also concurs with the description of FT consumers of Martin [24], in which they seem to be mostly young, professional-looking, artistic or students. On the other hand, our findings disagree with other studies performed in the SF sector, which describe that typical SF consumers are young, single, and unskilled workers with low educational level [25,26,27,28,29]. 

The results also revealed that most of the consumers were married, without children and employed, with one in four earning at least US $1225 (R $4770). Given that the Brazilian Institute of Geography and Statistics (IBGE) [30] puts the average per capita household monthly income in Brazil at approximately US $317 (R $1268), the average income of these consumers is at least 3-fold higher than the national average income. Additionally, data from the Pesquisa de Orçamentos Familiares (POF—Household Budget Survey), also carried out by IBGE, show that 31% of food purchase is spent with food outside of the home; however, there is no record of the expenditure with SF [3].

Occupational and household income play an important role in affecting consumers’ food spending habits [31]. Purchasing decisions and buying behavior are influenced by purchasing power and employment status. According to Warde and Martens [32], the frequency with which people eat out seems to be strongly associated with their social–demographic position; that is, those with higher incomes, higher educational levels, and couples without children tend to eat outside of the home more often. Even though people from various socioeconomic classes consume SF, employed consumers with high incomes are more likely to patronize fast food purchasing when compared to those unemployed and with low income, as some studies have suggested [27,33]. The findings of our study concur with those premises and probably reflect a distancing of the FT sector from the SF sector, even though the latter, in its essence, is integrated into the former. This differentiation can be explained by the features of FTs related to their production process and product presentation. The diversified and elaborated menu of FTs, including the quality of their ingredients and products, presented in an informal environment and at affordable prices than brick and mortar establishments, tend to attract young adults, married or not, who are not committed to providing a healthy and nutritional meal to their children.

The findings concerning the FT consumer profile of the current study also concur with the target population of a study conducted by Yoon and Chung [16]: Millennials. Millennials, who were born between 1980 and 2000, tend to eat out twice as frequently as the rest of the population and have more disposable income than that of the previous generation, which makes them the most powerful consumer group in the food service industry. In comparison to other generations, millennial consumers are considered more spontaneous and prone to adventure and novelty. These features are reflected in millennials’ food purchasing habits, affecting their choices and preferences. FTs are appealing to consumers due to their convenience, accessibility, taste, and their hedonic value [34]. Since FTs offer an innovative and interactive environment, with an authentic and gourmet cuisine at affordable prices, millennials are the most likely patrons and a powerful driver of the FT business.

Concerning FT consumers’ choices and preferences, most consumers eat from FTs once or twice a week (96%), near home (74%). It was also observed that consumers usually choose to eat in (67%) and during nighttime (96%), accompanied by family (66%) and friends (17%). The consumption of foods represents a mark of identity and social status, since a meal can be recognized as a social bond and represents a moment of leisure or celebration [35]. The results of this research well represent this sociocultural meaning of food consumers’ behavior. Further, eating out is provides consumers with the opportunity to satisfy their hunger and need for convenience, time saving, as well as pleasure, entertainment, social interactions, and mood transformation [36]. More than consuming nutrients, consumers dining in FTs accompanied by family and friends are consuming experiences, taste, and pleasure, as well as establishing group bonds. This is consistent with previous research presenting that FT consumers are involved in FT consumption primarily for hedonic reasons such as fun, excitement, and emotional worth [34]. In addition, the considerable change in the structure and function of the family, with more women in the working force and more people working longer hours, has contributed to the global nutrition transition [37] and, therefore, to the increasing consumption of fast food. Similarly, in Brazil, eating out has become common practice for a wide range of the population, especially urban, due to the industrial expansion and the participation of the female work force [38]. During this study, FTs were conveniently situated near living areas, where they provided a source of convenient food. 

Considering that eating out is considered a social event for FT consumers, FTs serve as a source of supplemental food—unlike traditional SF activity, which can be an integral or substantial part of the whole diet of consumers. The Brazilian eating pattern usually includes the consumption of three main meals (breakfast, lunch, and dinner), with lunch and dinner being marked by the presence of vegetables, rice, beans, fruits, and animal and vegetable protein sources. However, the habit of skipping or replacing dinner with less complex alternatives, such as snacks or fast foods, has been frequently observed in some families and individuals, especially younger ones [39]. It may help to explain the fact that hamburgers and sandwiches were the most popular food among consumers (72%), followed by pizza and pasta (10%). In Brazil, sandwiches are among one of the most consumed food outside of the home, corresponding to 9.61% of the per capita monthly expenditure [3]. In our study, the per capita expenditure FTs lies between US $5.14 to US $7.71 for more than 70% of consumers. 

Another possible explanation for the popularity of hamburgers and sandwiches is that health considerations did not seem to be the main factor driving consumers’ choices. According to Asp [40], psychological factors are among the strongest determinants of food choices, including food preferences and food likes and dislikes, since they may serve as an indicator of the amount of satisfaction one anticipates from eating a food. Regarding the reasons to choose a FT, only one consumer (0.7%) reported that nutritional value was the most important reason to choose a FT. The minor importance of the nutritional value while consuming food was also identified in a consumer study of Kabir et al. [41]. Although consumers perceive the nutritional aspects of fast foods, they often ignore those aspects in practice [42]. This indifference may be a possible reflection of the underestimation of the calorie content of their meals.

On the other hand, almost a third (30%) of consumers reported that taste was the most important reason to choose a FT, followed by accessibility/convenience (23%). These results are in harmony with findings of previous studies, which show that taste/flavor and convenience were ranked as critical factors that induced them to buy fast food [41,43,44]. While taste in a known influencing attribute on eating choices, the prominence of convenience is a reflection of the modern lifestyle pattern [1]. At the same time, poor vehicle hygiene (30%; *n* = 41) and poor food hygiene (20%; *n* = 28) were the main points assigned by consumers for not opting for a FT, confirming that hygiene is a criterion for high-income people that influences the consumption of fast food [45].

The food safety importance perception scores significantly differed (*p* < 0.05) with age, with the youngest age group (≤30) presenting the highest food safety importance perception scores and the oldest consumers (≥50) the lowest scores. This could have been a result of the fact that consumers today are more concerned about food quality and safety and also expect their food to be safe, wholesome, and tasty [46,47]. Moreover, the dissemination of public policies and resolutions, as well as social media, have an essential role in the improvement of consumer awareness on food safety, which is more powerfully experienced by the younger generation. Significant differences in food safety importance perception scores were also observed regarding the presence of children, with consumers without children obtaining higher food safety importance perception scores. This finding is alarming because young children are particularly susceptible to foodborne pathogens and, therefore, more at risk for FBDs due to their immature immune system. According to the World Health Organization (WHO), children under five account for almost one-third of the death rates of FBDs [9]. It is noteworthy to mention that consumers with children are mostly inserted in the oldest age group, with 49% belonging to the 31–50 age group and 37% belonging to the ≥50 age group, which may imply an indirect relation to the age group and, therefore, to lower food safety importance perception scores. 

On the other hand, no significant difference (*p* > 0.05) occurred in food safety importance perception for food safety training, level of education, gender or marital status. Although a statistical difference was expected for food safety training, it was not observed, probably due to the small sample of trained consumers. Although a statistical difference was also expected to gender, it was not observed in this study. Previous studies have shown that females have higher awareness of food safety [48,49], since they are mostly involved in food preparation, food handling, and in providing food for households [50]. As to the level of education, no significant difference was expected, since food safety is a specific content only covered by some graduate and postgraduate programs, as well as professional qualification courses on this subject, which depend on one’s occupation—which is, for instance, the case for food handling professionals. The absence of a significant difference regarding marital status may be a particularity of the sample of this study.

Regarding food safety importance perception, at least 85% of consumers demonstrated a high level of concern (very or extremely important) regarding questions 1 to 4—the usage of gloves, masks, caps and the existence of a hand sink for handwashing. The usage of caps by food handlers is mandatory, according to Brazilian regulation [51]. On the other hand, the usage of gloves must be done under specific criteria, following perfect hand hygiene and cleanliness conditions—which does not exempt food handlers from thoroughly washing their hands. The usage of masks, in turn, is not regulated by the federal sanitary legislation. Therefore, the fact of considering the usage of gloves, masks, caps, and handwashing as equally important is of major concern. First, it indicates a lack of understanding and knowledge by consumers, since those are distinct accessories, with distinct importance and usage modes, which when used incorrectly may cause more harm than good. Moreover, it demonstrates a possible false sense of security and a mistaken evaluation of the FTs by consumers, as if the use of all these accessories could represent a risk control measure against inadequate handling. Finally, attributing equal importance to the mentioned food handling apparel characterizes consumers’ illusion of control and social desirability bias. Weinstein [52] explains that the individual has a need for control over situations, so believing that they have control leads them to underestimate the associated hazards by identifying them as low risk or, in this case, of extremely importance for food safety. The occurrence of a social desirability bias, in turn, refers to the tendency of respondents to give socially desirable responses in such a way as to be viewed favorably by others [53]. This tendency can be expressed by overreporting and overestimating a socially desirable behavior. Since the usage of gloves, masks, and caps are a common practice in most food business and is usually associated with hygiene and cleanliness, consumers may have felt pressured to confirm the importance of their use, characterizing an overreported behavior. Asking participants to express their perception, in terms of stating how important the use of accessories which are frequently linked to adequate food hygiene and control was may also have led to participants being more likely to attach great importance to these accessories than they may otherwise have been, thus characterizing an overestimation behavior. However, as all FT consumers interviewed have been equally affected by this bias, the statistical differences found in this study could still be attributed to the statements previously discussed.

Most consumers also reported a high concern degree in questions 7 and 8, which are related to the correct usage of waste collectors and the absence of pest and vectors in the preparation area. However, a high proportion of consumers expressed indifference and little or no concern regarding other relevant food safety issues. In question 5, the fact that more than a third of the consumers are indifferent and 11% showed little or no concern about the usage of adornments or jewelry by the food handler demonstrates their lack of knowledge concerning the potential contamination of food by these personal objects. Adornments and jewelry represent potential physical or biological contamination, once they can fall over the food or accumulate dirt and microorganisms in their surfaces and become a source of cross-contamination [54]. 

In question 6, regarding the exclusive handling of money by the cashier, almost 15% of the consumers reported that either they were indifferent toward it or it had little or no importance, ignoring the fact that coins and currency notes could represent a potential cause of FBDs. Due to the survival of microorganisms of concern, money is an often-overlooked enteric disease reservoir that can serve as a vehicle for transmission of disease [55]. A significant proportion of consumers also disregarded the importance of critical temperatures of hot or cold ready-to-eat foods when they expressed indifference and little or no concern in questions 9 and 10. Temperature is frequently the critical control point of the production process. Therefore, if inadequately performed, temperature control may lead to the proliferation of microbial hazards and, consequently, to FBDs [56].

The findings of this research demonstrate that consumers are highly educated, earn larger incomes than the national average, and are involved in FT consumption primarily to hedonic reasons. Further, although most consumers reported considering food hygiene and vendors’ personal hygiene as important and the mean score indicated a high level of perceived importance, there is limited awareness of food safety by consumers, since they usually attached significance to what they can see and disregarded the importance of food temperature—and hence failed to assess conditions that may cause FBDs. According to Ergönül, only a few consumers who usually express concern on food safety appear to be changing their food buying and consumption behaviors given their concerns [57], which is also a serious concern. Therefore, food safety education for consumers is fundamental and should provide effective knowledge of food safety issues, targeted toward changing behaviors most likely to result in FBDs [58].

Recognition of personal responsibility for food safety should be considered a prerequisite to implementing appropriate food safety behaviors [59], that is, it may be necessary to increase perceived personal responsibility for food safety before attempting to promote behavioral changes. Since unrealistic optimism may seriously hinder efforts to promote risk-reducing behaviors [52], intervention strategies should focus on preventative and risk-reducing behaviors by enhancing consumers’ perceived vulnerability to FBDs. Parallelly, expansion of food-related disciplines to the basic education system should be considered, aiming to increase consumers’ food safety knowledge and awareness about their critical role in ensuring food safety, since uninformed consumers are more susceptible to misinformation [59]. Additionally, educational procedures and processes should be conducted by mass media—including the internet and social media—which has a major influence on consumer perception and should serve as a sharing environment of consumers’ experience and concern, a public communication channel to FBD outbreaks reports and an assistance tool for health departments for the improvement of sanitary inspections and control. Customer concern and awareness of food safety may be considered a driving force to create a better food hygiene environment, since an informed and educated public capable of choosing safe establishments and demand for hygiene will play an active role in selecting food service options able to deliver high food safety standards [60,61].

### Limitations

There are several potential limitations in this study. The small sample size and its convenient nature mean that the data presented here may not accurately represent the FT population of the Federal District or other regions of Brazil. A second limitation of this research is the fact that it distributed a survey with consumers in the dining area of FTs. Thus, responses could have bias compared to conducting surveys in other sites of the city/country. Further, since this is a cross-sectional survey, we recommend longitudinal studies to obtain more in-depth consumer information, such as the social and psychological factors driving FT consumers’ attitudes and behavioral intentions. Another limitation of this research is that possibly other variables—such as past or regular FT dining experience and/or personality features—might reflect differences in the food safety importance perception, increasing or reducing consumers’ level of risks and benefits perception toward FT consumption. Additionally, the running of face-to-face interviews considering consumers’ self-reported food safety importance perception could have contributed to the occurrence of a social desirability bias, which should be assessed in further investigations. Considering the mentioned differences among FT consumers could provide better understanding of FT consumption in future studies, as well as consumers’ attitudes and behavioral intentions. Future research should also consider the running of interviews with FT consumers in other sites of Brazil to identify possible generalizations, as well as other interview methods which eliminate interviewer effects in order to minimize the degree of social desirability bias. Despite these limitations, the interviews with FT consumers produced consistent information, as presented in the current study.

## 5. Conclusions

This is the first study to evaluate and report the FT consumers’ profile, choices, preferences, and food safety importance perception in Brazil. The findings of this study revealed that most FT consumers are highly educated and possess a favorable financial condition, but they also indicate that there remains the need for consumers to comprehend their role in the food supply chain. As consumers are the end users of food products, it is also important that they be aware of that hazards or critical points and conditions that may lead to FBDs, thus preventing the occurrence of the phenomenon of unrealistic optimism or the illusion of control. The data provided in this study are of significant concern since they can provide insight into the development of effective strategies to improve consumers’ awareness and safety of FTs in Brazil.

## Figures and Tables

**Table 1:** Sociodemographic variables and their association with food safety importance perception scores of food truck consumers studied in the Federal District, Brazil (*n* = 133).

Characteristic Evaluated	Response	Frequency (%)	Mean Score (SD)	*p* Value
Age	≤30	44 (33.1)	1.80 (0.48) ^A^	0.026
	31–50	72 (54.1)	1.66 (0.46) ^AB^	
	>50	17 (12.8)	1.46 (0.31) ^B^	
Gender	Male	59 (44.4)	1.74 (0.48) ^A^	0.192
	Female	74 (55.6)	1.64 (0.45) ^A^	
Marital status	With Companion (Married)	67 (50.4)	1.65 (0.48) ^A^	0.481
	Without Companion (Single/Divorced/Widowed)	66 (49.6)	1.71 (0.45) ^A^	
Children	Yes	90 (67.7)	1.77 (0.48) ^A^	0.001
	No	43 (32.3)	1.50 (0.36) ^B^	
Level of education	Secondary (High School)	26 (19.5)	1.67 (0.40) ^A^	0.652
	Tertiary (Graduate)	62 (46.6)	1.72 (0.49) ^A^	
	Quaternary (Postgraduate)	45 (33.8)	1.64 (0.47) ^A^	
Food safety training	Yes	16 (12)	1.53 (0.45) ^A^	0.166
	No	117 (88)	1.70 (0.46) ^A^	
Occupation status	Employed	118 (88.7)	-	-
	Unemployed or Retired	15 (11.3)	-	-
Monthly income (minimum wage; US $)	Not declared	2 (1.5)	-	-
	No income	4 (3.0)	-	-
	≤4 (US $980)	31 (23.3)	-	-
	5–8 (US $1225–1960)	36 (27.1)	-	-
	≥9 (US $2206)	64 (48.1)	-	-

SD: Standard deviation; ^A, B^ Groups with the same letters do not differ significantly.

**Table 2:** Choices and preferences of food truck consumers studied in the Federal District, Brazil (*n* = 133).

Item Evaluated	Response	Frequency	Percentage
Frequency of consumption (per week)	1–2 times	127	95.5
	3–4 times	5	3.8
	≥5 times	1	0.7
Place of consumption	Near home	100	75.2
	Near work	13	9.8
	Near university	1	0.7
	Other	19	14.3
Eating service	Eat-in	89	67
	Takeout	24	18
	Both	20	15
Time of consumption	Daytime	5	3.8
	Nighttime	128	96.2
Company	Family	90	67.7
	Friends	20	15
	Alone	23	17.3
Preferred type of food	Hamburgers and sandwiches	95	71.4
	Pizza and pasta	14	10.5
	Barbecue	7	5.3
	Meat and fish	4	3
	Other	13	9.8
Average expenditure on food (US $/per capita/per purchase)	<US $2.57	2	1.5
	US $2.57–5.14	13	9.8
	US $5.14–7.71	55	41.3
	US $7.71–10.27	42	31.6
	US $10.27–12.84	13	9.8
	>US $12.85	8	6
Reason to choose a food truck	Affordable	9	6.8
	Saving time	10	7.5
	Accessibility/Convenience	31	23.4
	Service quality	14	10.5
	Variety of options	14	10.5
	Taste	40	30.1
	Possibility to eat at any time	4	3
	Food hygiene	2	1.5
	Nutritional value	1	0.7
	Entertainment	8	6
Reason not to choose a food truck	Poor vehicle hygiene	41	30.9
	Long lines	27	20.3
	Insufficient number of vendors	1	0.7
	Solo dining	5	3.8
	Poor nutritional value	16	12
	Limited options	17	12.8
	Poor food hygiene	26	19.5
Do you consider food hygiene when eating from food trucks?	Always	104	78.2
	Most of the times	13	9.8
	Sometimes	9	6.8
	Rarely	6	4.5
	Never	1	0.7
Do you consider vendors’ personal hygiene when eating from food trucks?	Always	111	83.4
	Most of the times	15	11.3
	Sometimes	5	3.8
	Rarely	2	1.5
	Never	0	0

**Table 3:** Food safety importance perception of food truck consumers studied in the Federal District, Brazil (*n* = 133).

Is It Important if…	Response	Frequency	Percentage
1. The food handler wears gloves?	Extremely important	84	63.20%
	Very Important	37	27.80%
	Indifferent	9	6.80%
	Slightly important	3	2.20%
	Not at all important	0	0%
2. The food handler wears a mask?	Extremely important	63	47.40%
	Very Important	50	37.60%
	Indifferent	13	9.80%
	Slightly important	4	3.00%
	Not at all important	3	2.20%
3. The food handler wears a hair covering (a hair net or a cap)?	Extremely important	93	69.90%
	Very Important	35	26.30%
	Indifferent	5	3.80%
	Slightly important	0	0%
	Not at all important	0	0%
4. There is a hand sink, with hand soap and paper towels available for the food handler for handwashing?	Extremely important	89	67.00%
	Very Important	31	23.30%
	Indifferent	11	8.20%
	Slightly important	0	0%
	Not at all important	2	1.50%
5. The food handler does not wear adornments or jewelry?	Extremely important	38	28.60%
	Very Important	33	24.80%
	Indifferent	47	35.30%
	Slightly important	10	7.50%
	Not at all important	5	3.80%
6. Money is exclusively handled by the cashier?	Extremely important	93	70.00%
	Very Important	21	15.80%
	Indifferent	12	9.00%
	Slightly important	4	3.00%
	Not at all important	3	2.20%
7. All waste collectors of the preparation area are capped?	Extremely important	80	60.10%
	Very Important	40	30.10%
	Indifferent	9	6.80%
	Slightly important	2	1.50%
	Not at all important	2	1.50%
8. There are no vectors or pests in the preparation area?	Extremely important	117	88.00%
	Very Important	15	11.30%
	Indifferent	1	0.70%
	Slightly important	0	0%
	Not at all important	0	0%
9. Hot food is served hot?	Extremely important	38	28.60%
	Very Important	45	33.80%
	Indifferent	36	27.10%
	Slightly important	11	8.30%
	Not at all important	3	2.20%
10. Cold food is served cold?	Extremely important	50	37.60%
	Very Important	47	35.30%
	Indifferent	21	15.80%
	Slightly important	9	6.80%
	Not at all important	6	4.50%

## Supplementary Materials

The following are available online at https://www.mdpi.com/2072-6643/11/5/1175/s1, S1: Food truck consumer questionnaire.
