# Supplementary material for: Brazilian Food Truck Consumers’ Profile, Choices, Preferences, and Food Safety Importance Perception
Source: Nutrients. 2019 May 25;11(5):1175. doi: 10.3390/nu11051175 (PMC6566650; doi:10.3390/nu11051175)
Supplement: Supplementary file 1 [file nutrients-11-01175-s001.pdf]

FOOD TRUCK CONSUMER QUESTIONNAIRE

Name:	Email/Phone:
Number/Year:	

SOCIO-DEMOGRAPHIC STATUS
**1. Age**
( ) ≤30<br>( ) 31-50<br>( ) >50
**2. Gender**
( ) Male<br>( ) Female
**3. Marital status**
( ) With Companion (Married)<br>( ) Without Companion (Single/Divorced/Widowed)
**4. Children**
( ) Yes<br>( ) No
**5. Level of education**
( ) Secondary (High School)<br>( ) Tertiary (Graduate)<br>( ) Quaternary (Postgraduate)
**6. Food safety training**
( ) Yes<br>( ) No
**7. Occupation status**
( ) Employed<br>( ) Unemployed or Retired
**8. Monthly income (minimum wage; US$)**
( ) Not declared<br>( ) No income<br>( ) ≤4 ((US$ 980)<br>( ) 5-8 (US$ 1,225 - 1,960)<br>( ) ≥9 (US$ 2,206)
**CHOICES AND PREFERENCES**
**1. Frequency of consumption (per week)**
( ) 1-2 times<br>( ) 3-4 times<br>( ) ≥5 times

**2. Place of consumption**
( ) Near home<br>( ) Near work<br>( ) Near university<br>( ) Other
**3. Eating service**
( ) Eat in<br>( ) Takeout<br>( ) Both
**4. Time of consumption**
( ) Daytime<br>( ) Nighttime
**5. Company**
( ) Family<br>( ) Friends<br>( ) Alone
**6. Preferred type of food**
( ) Hamburgers and sandwiches<br>( ) Pizza and pasta<br>( ) Barbecue<br>( ) Meat and fish<br>( ) Other
**7. Average expenditure on food (US$/per capita/per purchase)**
( ) <US$ 2.57<br>( ) US$ 2.57 - 5.14<br>( ) US$ 5.14 - 7.71<br>( ) US$ 7.71 - 10.27<br>( ) US$ 10.27 - 12.84<br>( ) > US$ 12.85
**8. Reason to choose a food truck**
( ) Affordable<br>( ) Saving time<br>( ) Accessibility/Convenience<br>( ) Service quality<br>( ) Variety of options<br>( ) Taste<br>( ) Possibility to eat at any time<br>( ) Food hygiene<br>( ) Nutritional value<br>( ) Entertainment
**9. Reason not to choose a food truck**
( ) Poor vehicle hygiene<br>( ) Long queues<br>( ) Insufficient number of vendors<br>( ) Solo dining<br>( ) Poor nutritional value

( ) Limited options
( ) Poor food hygiene

10. Do you consider food hygiene when eating from food trucks?

( ) Always
( ) Most of the times
( ) Sometimes
( ) Rarely
( ) Never

11. Do you consider vendors' personal hygiene when eating from food trucks?

( ) Always
( ) Most of the times
( ) Sometimes
( ) Rarely
( ) Never

FOOD SAFETY IMPORTANCE PERCEPTION

1. Is it important if the food handler wears gloves?

( ) Extremely important
( ) Very Important
( ) Indifferent
( ) Slightly important
( ) Not at all important

2. Is it important if the food handler wears a mask?

( ) Extremely important
( ) Very Important
( ) Indifferent
( ) Slightly important
( ) Not at all important

3. Is it important if the food handler wears a hair covering (a hair net or a cap)?

( ) Extremely important
( ) Very Important
( ) Indifferent
( ) Slightly important
( ) Not at all important

4. Is it important if there is a hand sink, with hand soap and paper towels available for the food handler for handwashing?

( ) Extremely important
( ) Very Important
( ) Indifferent
( ) Slightly important
( ) Not at all important

5. Is it important if the food handler does not wear adornments or jewelry?

( ) Extremely important
( ) Very Important
( ) Indifferent

( ) Slightly important
( ) Not at all important

6. Is it important if money is exclusively handled by the cashier?

( ) Extremely important
( ) Very Important
( ) Indifferent
( ) Slightly important
( ) Not at all important

7. Is it important if all waste collectors of the preparation area are capped?

( ) Extremely important
( ) Very Important
( ) Indifferent
( ) Slightly important
( ) Not at all important

8. Is it important if there are no vectors or pests in the preparation area?

( ) Extremely important
( ) Very Important
( ) Indifferent
( ) Slightly important
( ) Not at all important

9. Is it important if hot food is served hot?

( ) Extremely important
( ) Very Important
( ) Indifferent
( ) Slightly important
( ) Not at all important

10. Is it important if cold food is served cold?

( ) Extremely important
( ) Very Important
( ) Indifferent
( ) Slightly important
( ) Not at all important
